# Impact of ultrasound-assisted enzymatic extraction on the structural and functional properties of *Tremella fuciformis* polysaccharide and its application in almond-based milk^☆^

**DOI:** 10.1016/j.ultsonch.2026.107897

**Published:** 2026-05-22

**Authors:** Furong Hou, Shasha Song, Jie Mi, Yansheng Wang, Hao Dong, Wenliang Wang

**Affiliations:** a Institute of Agro-Food Sciences and Technology, Shandong Academy of Agricultural Sciences, Jinan 250100, Shandong, China; b College of Life Sciences, Shandong Agricultural University, Taian, Shandong, China; c Rizhao Meijia Keyuan Food Co., Ltd, Rizhao, Shandong, China

**Keywords:** *Tremella fuciformis* polysaccharides, Ultrasound-assisted extraction, Structural characteristics, Functional properties, Almond-based milk

## Abstract

Ultrasound, when combined with enzymatic hydrolysis, distinctly influences the macromolecular structure of *Tremella fuciformis* polysaccharide, yielding a polysaccharide with improved functional properties. To comprehensively evaluate these attributes and validate its practical relevance, three polysaccharide samples were systematically compared: those produced by ultrasound-assisted enzymatic extraction (UAEP), ultrasound-alone extraction (UEP), and enzyme-alone extraction (EEP). It was found that UAEP exhibited significantly higher yield (1.5- and 2.5-fold greater than UEP and EEP, respectively) while concurrently reducing the weight-average molecular weight to 955 kDa, notably lower than UEP (1070 kDa) and EEP (1770 kDa). Structural analyses revealed that UAEP possessed a more porous and looser microstructure, although its monosaccharide composition and triple-helix conformation remained similar to other samples. Functionally, UAEP exhibited markedly higher emulsifying stability alongside lower apparent viscosity and superior thermal stability. Rheological characterization confirmed its pseudoplastic shear-thinning behavior with distinct viscoelasticity. Multivariate statistical analyses, including principal component analysis and correlation analysis, quantitatively established that key structural parameters, particularly molecular weight, protein and uronic acid content, were closely linked to its enhanced emulsifying performance. To validate its practical utility, UAEP was successfully incorporated into almond-based milk, where it achieved significantly enhanced physical stability (25.11%) and textural properties (consistency: 55.81 g·sec, cohesiveness: −9.84 g) comparable to a commercial walnut protein-based milk (physical stability: 12.38%, consistency: 56.22 g·sec, cohesiveness: −10.09 g). These findings demonstrate that ultrasound-assisted enzymatic extraction not only improves polysaccharide yield but also modulates its macromolecular structure, offering a viable approach to obtain multifunctional hydrocolloids for plant-based food applications.

## Graphical abstract

**Figure:**
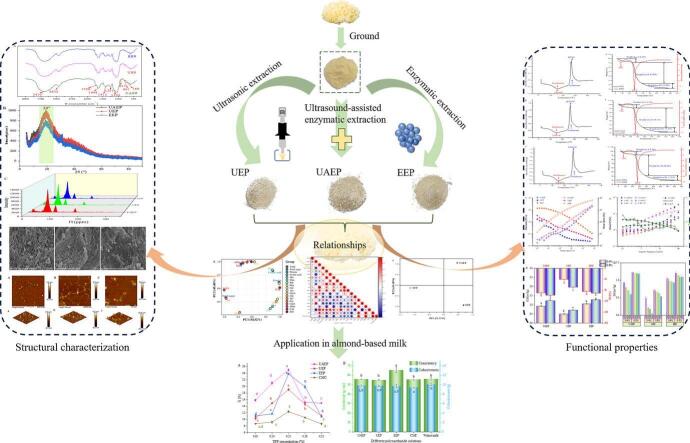

## 1 Introduction

*Tremella fuciformis* is an edible and medicinal macrofungus that has gained extensive cultivation in Asian countries due to its appealing flavor and remarkable nutritional value [1]. Among its bioactive components, *Tremella fuciformis* polysaccharides (TFP), accounting for approximately 60–70% of the dry weight, have garnered substantial attention due to its remarkable biological properties, including hypolipidemic, antioxidant and anti-inflammation activity [2], [3], alongside promising functional attributes, such as water holding capacity, emulsifying activity and swelling ability [4].

Generally, TFP is an anionic heteropolysaccharides with the weight-average molecular weight (M_w_) ranging from several hundred thousand to tens of millions of Dalton [5]. It is characterized by a linear backbone of α‑(1 → 3)-linked D‑mannose residues and side branches composed of D‑glucuronic acid, D‑xylose, and L‑fucose, which are linked via α‑(1 → 2) bonds at positions 2, 4, and 6 [6]. These structural features significantly affect the functional properties. For instance, it has been confirmed that an increase in M_w_ of TFP is correlated with stronger intermolecular interactions and higher viscosity in aqueous systems, contributing to desirable textural outcomes such as lubricity in food products and a moist sensation in skincare applications [7]. Additionally, the high uronic acid content of TFP is associated with enhanced thermal stability, further underscoring its potential as a functional hydrocolloid [8].

Extraction is the primary and critical step in the study of polysaccharides, as the method employed directly influences key parameters including yield, molecular weight, monosaccharide composition, and physicochemical properties. The structural characteristics are largely determined by the extraction process, in turn govern their functional properties [5]. While the impact of extraction on structural and physicochemical characteristics of TFP has been explored in several studies, most have focused on conventional single‑method approaches such as cold/hot water, acid/alkali, or enzymatic extraction [8], [9], [10]. These methods often involve inherent trade-offs between extraction efficiency, environmental impact, and cost. For instance, hot‑water extraction is simple and low‑cost but inefficient even under prolonged heating; acid/alkali treatments improve yield but risk glycosidic bond cleavage and environmental concerns; enzymatic methods offer mild conditions and good polysaccharide preservation but are often time‑consuming and expensive [5].

Given these limitations, integrated extraction strategies have emerged as a promising alternative. It has been documented that combining extraction methods can enhance the yield of Tremella polysaccharides by 3–4 times compared to single‑method approaches [7]. In recent years, ultrasound has gained prominence as an environmentally benign, non‑thermal processing technology for polysaccharide extraction. Combined extraction approaches, especially those assisted by ultrasound, have demonstrated superior performance over conventional single‑method techniques, providing higher polysaccharide yields while better preserving bioactivity. For instance, Li et al. [2] employed an ultrasonic‑enzymatic‑assisted ethanol precipitation method to extract TFP from *Tremella fuciformis* spore fermentation broth, demonstrating its superiority over single‑method approaches in terms of yield and bioactivity. This study, however, have primarily focused on process optimization and bioactivity assessment. A systematic evaluation of how different extraction methods, particularly the combined ultrasound-enzymatic approach, affect the detailed structural parameters of TFP and, in turn, how these structural differences correlate with a range of physicochemical functionalities (e.g., emulsifying, rheological, and thermal properties) has been less explored, particularly with respect to their application within real food systems.

To address this, the present study systematically compares TFP extracted by ultrasound-assisted enzymatic, ultrasound-alone, and enzyme-alone methods. The objective is to assess the influence of these extraction methods on key structural characteristics and to evaluate the resulting functional performance. Multivariate statistical analyses are used to examine correlations between structural features and functional properties. Finally, the practical application potential of the TFPs is tested in a plant-based milk system, linking the laboratory findings to a relevant food model.

## 2 Materials and methods

### 2.1 Materials and reagents

*Tremella fuciformis,* almonds and the commercial protein beverage were purchased from a local supermarket in Jinan (Shandong, China). Cellulase was purchased from Shanghai Macklin Biochemical Technology Co., Ltd. (Shanghai, China). All other reagents were of analytical grade.

### 2.2 Preparation of different TFP samples

*Tremella fuciformis* was dried at 50°C and ground into fine powder (100-mesh). Then it was extracted by ultrasound-assisted enzymatic extraction with a probe ultrasonic processor (SCIENTZ-IID, Ningbo Scientz Biotechnology Co., Ltd, Zhejiang, China) operating at frequency of 20 kHz. The extraction temperature was controlled by a low-temperature thermostatic water bath (DC-1015, Ningbo Scientz Biotechnology Co., Ltd., Zhejiang, China). The extraction conditions were selected as follows: a solid-to-liquid ratio of 1:90 (g/mL), pH 5.0, cellulase (10,000 U/g, Shanghai Macklin Biochemical Technology Co., Ltd., China) concentration of 3% (w/v), ultrasonic power of 600 W (actual power 559.46 W, measured by calorimetric method [11]), temperature of 45℃, time of 40 min, and cellulase concentration of 3% (w/v). After the extraction, the enzymes were inactivated in boiling water for 10 min, and then cooled and centrifuged at 4000 r/min for 15 min to collect the supernatant. Then it was precipitated with 95% (v/v) ethanol at a 4:1 (v/v) ratio to the supernatant, followed by incubation at 4℃ for 4 h. After centrifugation (4000 r/min, 15 min), the precipitate was dialyzed against distilled water using MD34 dialysis bag (M_w_ of 8–14 kDa) for 48 h, then lyophilized for 36 h. The TFP yield was calculated as follows: Yield (%) = (Weight of dried TFP/Weight of *Tremella fuciformis* powder) × 100%. The polysaccharide extracted via the ultrasound-assisted enzymatic method was designated as UAEP. For comparison, products were obtained using ultrasound alone (under identical conditions but without enzyme), denoted as UEP, and using enzymatic hydrolysis alone (at 50°C for 150 min with 3% (w/v) enzyme concentration), denoted as EEP.

### 2.3 The physicochemical characteristics of TFPs

#### 2.3.1 Determination of total sugar, protein, and uronic acid contents

The total sugar, protein, and uronic acid contents were determined using the phenol‑sulfuric acid method, the Bradford assay, and the sulfuric acid‑carbazole method, respectively.

#### 2.3.2 Water contact angle measurement

An optical contact angle meter (Dataphysics OCA20, DataPhysics Instruments GmbH, Stuttgart, Germany) is applied to measure the water contact angles (θ) of TFP powders. Approximately 0.5 g of TFP powder was compressed into a tablet. Subsequently, a 4 μL droplet of deionized water was gently deposited onto the tablet surface using a syringe, and a sequence of 400 frames was captured immediately by a high-speed camera.

### 2.4 The structural characteristics of TFPs

#### 2.4.1 Monosaccharide composition

Samples (5 mg) were hydrolyzed with 4.0 mL of 2 mol/L trifluoroacetic acid in a 10 mL ampoule at 120°C for 2 h. After hydrolysis, the product was dried under nitrogen, rinsed with methanol, and diluted with ultrapure water. An aliquot (100 μL) of the derivatization solution (100 μL) was then mixed with 100 μL of NaOH (0.6 mol/L) and 100 μL of PMP (0.5 mol/L) in a 5 mL EP tube, and incubated at 70℃ for 100 min. After cooling to room temperature, the mixture was neutralized with HCl (0.3 mol/L, 200 μL), then evaporated to dryness at 50℃. The residue was subsequently extracted with chloroform, and the resulting solution was filtered through a 0.45 μm membrane prior to injection. Monosaccharide composition was analyzed using the following monosaccharide standards: mannose (Man), glucose (Glu), xylose (Xyl), fucose (Fuc), glucuronic acid (GluA), galactose (Gal), arabinose (Ara), ribose (Rib), rhamnose (Rha) and galacturonic acid (GalA) (all ≥ 98% purity, Sigma-Aldrich, USA).

HPLC analysis was carried out on a LC-20AD HPLC system (Shimadzu, Japan) equipped with an Xtimate C18 column (200 × 4.6 mm, 5 μm). The column temperature was 30℃ with a flow rate of 1.0 mL/min and an injection volume of 10 μ L. The mobile phase consisted of 0.01 M KH_2_PO_4_ (pH 6.9) and acetonitrile (83:17, v:v), and the detection wavelength was set to 245 nm.

#### 2.4.2 Molecular weight

Molecular parameters were determined using an Agilent 1260 gel permeation chromatography system (Agilent Technologies Inc., USA) equipped with a PL aquagel-OH MIXED-H (330 × 7.5 mm, 8 μm). Prior to injection, samples were dialyzed and filtered through a 0.45 μm membrane. The mobile phase was 0.1 mol/L Na_2_NO_3_ containing 0.02% NaN_3_, with a flow rate of 1.0 mL/min, an injection volume of 200 μL, and a column temperature of 40°C.

#### 2.4.3 The Fourier transform-infrared (FTIR) spectra

TFP samples (2 mg) were mixed with 0.5 g of KBr and compressed into a pellet. The spectra were acquired using a Nicolet 380 FTIR spectrometer (Thermo Fisher Scientific, MA, USA) at a resolution of 4 cm^−1^ in the wavenumber range of 4000–400 cm^−1^.

#### 2.4.4 X-ray diffraction (XRD)

XRD analysis was performed on a SmartLab9 Kw diffractometer (Rigaku, Japan) with Cu Kα radiation (λ = 0.15405 nm, 40 kV, 40 mA). The scanning range was 5–90° (2θ) at a scanning speed of 8°/min and a step size of 0.08°.

#### 2.4.5 Nuclear magnetic resonance (NMR)

TFP samples (∼300 mg) were subjected to two rounds of D_2_O exchange and then dispersed in D_2_O. Spectra were acquired on a 400 MHz NMR spectrometer (Advance Ⅲ HD, Bruker BioSpin GmbH, Switzerland) at 25°C.

#### 2.4.6 Scanning electron microscope (SEM)

Freeze-dried TFP samples were sputter-coated with gold under vacuum and then examined by SEM (S4800 Hitachi Corp., Japan) at an acceleration voltage of 15 kV to observe their surface morphologies.

#### 2.4.7 Atomic-force microscopy (AFM)

TFP samples were dissolved to a final concentration of 1 mg/mL and subjected to ultrasonication for 5 min. An aliquot of the dispersed solution was then dropped onto a freshly cleaved mica surface and allowed to air-dry. The microstructure of the samples was characterized by a Bruker Dimension FastScan atomic force microscope (Bruker Corporation, Germany).

#### 2.4.8 Congo red experiment

TFP solutions (1 mg/mL, 2 mL) were mixed with an equal volume of Congo Red solution (100 µmol/L, 2 mL), followed by the addition of NaOH at various concentrations (0–0.5 mol/L, 4 mL). The maximum absorption wavelength was recorded using a UV spectrophotometer (UV-6100, Shanghai Yuanxi Instrument Co., Ltd, China) over the range of 400–600 nm.

### 2.5 The functional properties of TFPs

#### 2.5.1 Thermal properties

Differential scanning calorimetry (DSC) analysis was performed on a Discovery DSC 250 calorimeter (TA Instruments, USA). TFP samples (approximately 4 mg) were sealed in aluminum crucible and heated from 30 to 450℃ at a rate of 10 ℃/min under a nitrogen flow of 50 mL/min, with an empty crucible as reference. Thermo-gravimetric analysis (TGA) was conducted using a simultaneous thermal analyzer (STA 449 F5, Netzsch-Gerätebau GmbH, Selb/Germany) over a temperature range of 30–700℃ at a heating rate of 10 ℃/min.

#### 2.5.2 Rheological measurements

Rheological measurements were performed on an HR20 Rheometer (TA Instruments, New Castle, USA) equipped with a 40 mm parallel plate. TFP solutions (2%, w/w) were analyzed at 25°C over a shear rate range of 0.01–600 s^−1^. The obtained data were subsequently fitted to the Power-Law model (Eq. (1)):

(1) $$\eta \;\text{= K}\gamma^{\text{n-1}}$$

where η is the viscosity (Pa·s), K is consistency coefficient (Pa·s^n^), γ is the shear rate (s^−1^), n is the flow behavior index.

The linear viscoelastic region (LVR) was determined by an oscillation amplitude strain sweep (01% to 100%). Dynamic viscoelastic properties were then measured within the LVR (strain of 1%) over an angular frequency range of 0.1–100 rad/s.

#### 2.5.3 Emulsifying properties

The emulsifying properties of TFP samples were measured by the turbidity method. Briefly, TFP solutions at concentrations of 0.4% and 0.8% (w/v, 20 mL) were mixed with palm oil (2 mL), and homogenized at 10,000 r/min for 1 min using a high-speed homogenizer (XHF-DY, Ningbo Scientz Biotechnology Co., Ningbo, China) to obtain emulsions. Afterwards, 100 μL of TFP emulsions were pipetted at 0 and 10 min and then diluted 100 times with 0.1% sodium dodecyl sulfate (SDS). The absorbance of the diluted samples was measured at 500 nm (A_500_) using a UV-6100 spectrophotometer (Shanghai Yuanyan Instrument Co., Ltd., Shanghai, China), with 0.1% SDS solution serving as the reference. The emulsifying activity (EAI) and emulsifying stability (ESI) were calculated by the following equations:

(2) $$\text{EAI (}{\text{m}}^{2}\text{/g) =}\;\frac{\text{4.606 ×}{\;\text{A}}_{\text{500}}}{\text{C × φ × 1000}}\;\text{× DF}$$

(3) $$\text{ESI}\;\left(\text{\%}\right)=\frac{{\text{A}}_{\text{10}}}{{\text{A}}_{\text{0}}}\;\text{× 100\%}$$

where DF is the dilution factor (100), C is the concentration of TFP (g/mL), and φ is the oil volumetric fraction. A_0_ and A_10_ are the absorbances at 0 min and 10 min, respectively.

### 2.6 The application of TFPs in almond-based milk

#### 2.6.1 Preparation of almond-based milk

Almonds were mixed with water at a ratio of 1:10 (m/v) and boiled for 5 min. The boiled almonds were immediately removed and cooled with cold water, then peeled. The peeled almonds were mixed with purified water at a ratio of 1:50 (m/v) and ground using a blender. The mixture was filtered through a 200-mesh sieve, and the filtrate was homogenized at 10,000 r/min for 1 min to obtain the almond milk.

#### 2.6.2 Determination of stability coefficient of almond-based milk

Different TFPs and carboxymethyl cellulose (CMC, a common commercial emulsifying stabilizer) samples were added to the pre-prepared almond-based milk at specified ratios (0.05%, 0.1%, 0.15%, 0.2%, 0.25%, w/v) to evaluate their effects on the stability of the emulsion system (room temperature). Then the samples were centrifuged at 4000 r/min for 10 min. The supernatant was collected, diluted 100-fold, and its absorbance (A_2_) was measured at 720 nm using a spectrophotometer. The stability coefficient (R) was calculated as the ratio of A_2_ to the absorbance before centrifugation (A_1_).

#### 2.6.3 Texture analysis

The consistency and cohesiveness of almond-based milk added with UAEP, UEP, EEP and CMC were determined using SMS TA.XT plus/50 texture analyzer (Beijing Weixun Chaoji Instrument Technology Co., Ltd) with an A/BE-d45 probe. The test parameters were as follows: pre-test speed of 1.00  mm/sec, test speed of 2.00  mm/sec, post-test speed of 10.00  mm/sec, displacement distance of 10  mm and compression force of 5.0  g.

### 2.7 Statistical analysis

All experiments were conducted in triplicate, with data presented as mean ± standard deviation (SD). Principal component analysis (PCA) and Pearson correlation analysis were carried out using SPSS 19.0 (IBM software, New York, USA) based on individual replicate measurements (n = 9, three replicates per sample) as exploratory tools. Statistical analysis was performed analyzed by one-way ANOVA supplemented with Tukey's post hoc test for multiple comparisons, with statistical significance defined as *p* < 0.05.

## 3 Results and discussion

### 3.1 The physicochemical characteristics TFPs

#### 3.1.1 Chemical composition

The chemical compositions of TFPs derived from different extraction methods are shown in Table 1. Notably, UAEP achieved a yield of 28.19%, which was significantly higher than those of UEP (18.79%) and EEP (11.23%), underscoring the positive contribution of ultrasound to TFP extraction. Ultrasonic treatment effectively disrupted cell wall structures, facilitating solvent penetration and polysaccharide dissolution, thereby enhancing the TFP release and yield [12]. The combination of ultrasound and enzyme not only emphasized the role of ultrasound but also improved the enzymatic efficiency in disrupting cell wall, degrading internal storage compounds, and releasing intracellular substances [13]. The M_w_ of all three TFPs ranged from 8 × 10^3^ to 3.43 × 10^6^ Da, in agreement with the range reported by Wu et al. [14]. Compared with EEP, both UEP and UAEP exhibited markedly lower M_w_ due to sonolysis and sonoenzymolysis, respectively [15]. Beyond the M_w_, the polydispersity index (PDI = M_w_/M_n_, where M_n_ is the number-average molecular weight), which reflects the homogeneity of polymer molecular weights, varied considerably among the three samples. As shown in Table 1, UEP showed a markedly lower PDI of 2.33 compared to EEP (5.51), indicating that ultrasonication partially homogenized the molecular weight distribution. Furthermore, UAEP yielded the lowest PDI of 1.97, indicating that the combination of ultrasound and enzymatic hydrolysis not only reduced the M_w_ but also produced a more uniform and narrowly distributed polysaccharide population. Regarding the content of total sugar and uronic acid, no significant differences were observed among these three samples. The difference in protein content might potentially contribute to their emulsifying properties. This speculation is supported by Sanchez et al. [16], who reported that the emulsifying activity of arabic gum was related to its protein portion, and removal of protein led to loss of emulsifying ability. However, direct evidence linking protein content to emulsifying performance in the present polysaccharide system is still lacking, and further studies are needed to confirm this relationship.

**Table 1:** Yields and physicochemical characteristics of TFP extracted by different methods.

	UAEP	UEP	EEP
Yield (%, w/w)	28.19 ± 0.42%^a^	18.79 ± 0.69^b^	11.23 ± 0.57^c^
Total sugar (%, w/w)	55.06 ± 0.18^a^	55.85 ± 0.66^a^	54.98 ± 0.24^a^
Total protein content (%, w/w)	3.30 ± 0.01^b^	2.89 ± 0.01^c^	3.41 ± 0.02^a^
Uronic acid (%, w/w)	18.35 ± 0.26^a^	17.61 ± 0.095^b^	18.13 ± 0.36^a,b^
M_w_ (Da)	(9.55 ± 0.32)^c^ × 10^5^	(1.07 ± 0.04)^b^ × 10^6^	(1.77 ± 0.06)^a^ × 10^6^
M_n_ (Da)	(4.85 ± 0.78)^b^ × 10^5^	(4.58 ± 0.29)^a^ × 10^5^	(3.21 ± 0.54)^b^ × 10^5^
Polydispersity (M_w_/M_n)_	1.97 ± 0.36^c^	2.33 ± 0.21^b^	5.51 ± 0.56^a^
Monosaccharide component (mol%)			
Man	41.94	37.01	40.39
Rib	0.39	0.45	0.63
Rha	0.17	0.15	0.15
GluA	2.89	2.84	3.30
GalA	0.21	0.31	0.15
Glu	19.65	24.17	22.51
Gal	1.49	1.32	2.12
Xyl	18.69	20.95	18.55
Ara	0.08	0.05	0.09
Fuc	14.47	12.76	12.12
Contact angle (°)	60.17 ± 2.31^a^	50.38 ± 1.56^b^	46.15 ± 2.09^c^

Note: different letters in the same row represent significant differences (*p <* 0.05).

M_w_: weight-average of molecular weight. M_n_: number-average of molecular weight.

UAEP: *Tremella fuciformis* polysaccharide (TFP) extracted by ultrasound-assisted enzymatic method, UEP: TFP extracted by ultrasound-alone method, EEP: TFP extracted by enzyme-alone method.

#### 3.1.2 The monosaccharide composition

It could be seen in Table 1, the three samples exhibited similar monosaccharide compositions regardless of the extraction method, although their molar ratios varied [17]. Overall, TFPs were predominantly composed of mannose, glucose, xylose, fucose, glucuronic acid and galactose. Among these, mannose was the most abundant monosaccharide, followed by glucose and xylose. Together, these three neutral sugars accounted for over 80% of the total monosaccharides. Given the predominance of mannose, it is likely that mannose forms the main chain of these polysaccharides, which is consistent with previous reports [18].

#### 3.1.3 Water contact angle of TFP powders

The contact angle is commonly used to characterize the surface wettability of materials, reflecting their hydrophilic or hydrophobic nature. As shown in Table 1, all of the TFPs exhibited contact angles (θ) below 90°, indicating hydrophilic surface properties [19]. This finding is consistent with the fact that TFP chains contain abundant hydroxyl groups. The slight differences in contact angles among UAEP, UEP, and EEP might be attributed to their distinct molecular structures (such as M_w_ and microstructure), which could affect the packing density and accessibility of hydrophilic groups at the tablet surface upon compression.

### 3.2 The structural characteristics of TFPs

#### 3.2.1 FTIR

**Fig. 1:**
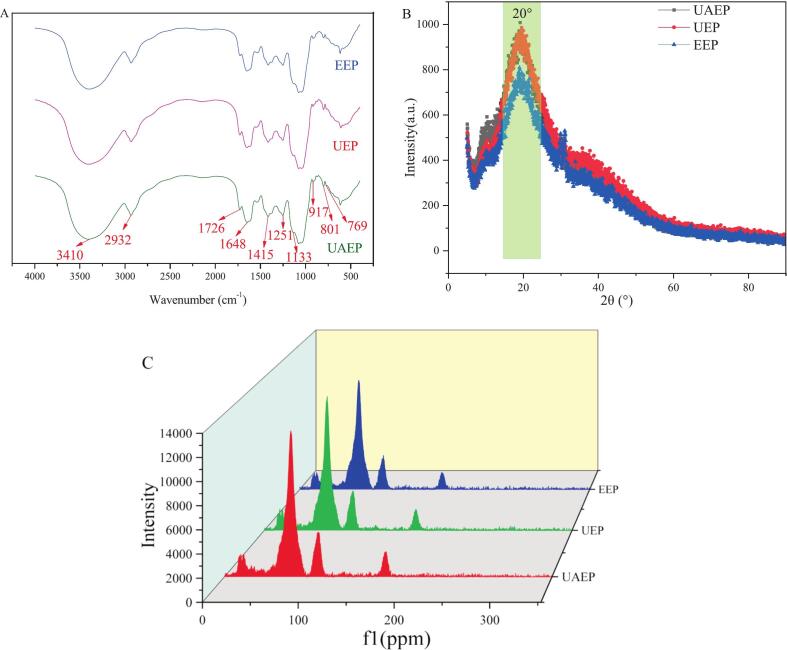
FTIR (A), XRD (B), ^13^C NMR (C) spectra of different TFPs (UAEP: *Tremella fuciformis* polysaccharide (TFP) extracted by ultrasound-assisted enzymatic method, UEP: TFP extracted by ultrasound-alone method, EEP: TFP extracted by enzyme-alone method).

As shown in Fig. 1(A), the characteristic absorption peaks were essentially identical across all samples, indicating that neither ultrasound nor enzyme treatment altered the main functional group structures, a finding consistent with Li et al. [2]. A prominent absorption band at approximately 3410 cm^−1^ and a weak peak at 2932 cm^−1^ were attributed to the stretching vibration of O-H and C-H of the sugar ring, respectively [20]. This presence of O-H groups indicated the hydrophilic nature of TFPs, as reflected by their low contact angles (Table 1). The absorption peak at around 1726 cm^−1^ and 1648 cm^−1^ corresponded to the C=O asymmetric stretching vibration in –COOH, confirming the presence of uronic acid. The signal at 1415 cm^−1^ was related to the C=O stretching vibration of the carboxyl group [9]. The peaks at 1251 cm^−1^ and 1133 cm^−1^ were assigned to C-H variable angle vibration and asymmetric C-O-C stretching of the sugar ring, respectively. The typical bands near 917 cm^−1^ and 769 cm^−1^ were associated with the existence of α-type pyranose and xylose [21]. A characteristic peak at 801 cm^−1^ suggested residual mannose, consistent with the monosaccharide composition analysis which identified mannose as the most abundant monosaccharide in TFPs. Collectively, the FTIR results confirmed that TFPs were mainly composed of mannose, glucose, xylose, fucose and glucuronic acid.

#### 3.2.2 XRD

XRD analysis was performed to examine the crystalline or amorphous nature of TFPs (Fig. 1(B)). Obvious broad diffraction peaks at around 2θ = 10° and 20° were observed in all three samples, indicating that all TFPs exhibited amorphous properties [22]. Notably, the broad peak at approximately 20° has been reported to reflect ordered molecular packing arising from intermolecular interactions, including hydrogen bonding [23]. Overall, the three XRD patterns were similar, indicating that the different extraction methods did not alter the amorphous structure of TFPs. As seen in Fig. 1(B), EEP exhibited the lowest peak intensity among the three samples, which might suggest a more disordered amorphous structure compared to UAEP and UEP [24].

#### 3.2.3 NMR

As shown in Fig. 1(C), the ^13^C NMR spectra of these three samples displayed similar signal patterns, suggesting that the main chemical structures would not be undermined after enzymolysis and sonoenzymolysis, in agreement with the FTIR and XRD results. The number of anomeric carbon signals observed in the resonance range of 95–110 ppm indicated that TFPs were composed of various types of monosaccharide residues. Signal in the range of 97–101 ppm implied the existence of α-configuration [25]. The signal at *δ*173.86 was assigned to the carboxyl group of GluA [26], and the peak at *δ*20.18 ppm corresponded to acetyl CH_3_ [27]. Signals below *δ*20 ppm implied the existence of Rha [28]. The strongest peak at 71.65 ppm was attributed to C-2 of α-Man, which corroborated the monosaccharide composition analysis showing Man as the predominant monosaccharide component of TFPs [25].

#### 3.2.4 SEM

**Fig. 2:**
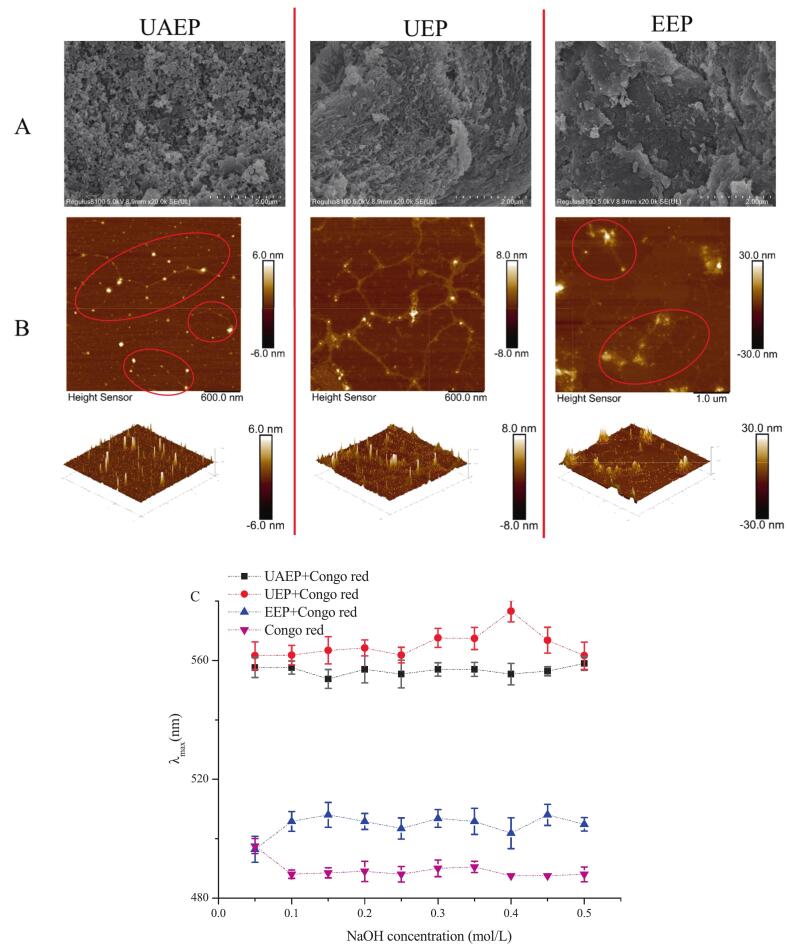
SEM (A), and AFM (B) images of different TFPs, Congo red experiment of TFPs (C).

As shown in Fig. 2(A), the three TFPs exhibited markedly different surface morphologies with respect to shape and size. UAEP displayed a honeycomb-like surface characterized by numerous small pores, grooves and rough fractures. UEP appeared as relatively large rough sheets or clumps with flat and densely distributed surface voids. In contrast, EEP exhibited relatively rough thin sheets composed of numerous flakes, forming a compact structure than UEP and UAEP. Similar polysaccharides characteristics have been documented by Li et al. [2]. During the ultrasonic process, cavitation effects disrupted the chain entanglements within the polysaccharide network, leading to the breakdown of the polymer structure. The combination of ultrasound and enzymes further facilitated this disrupting, resulting in a more porous and loose structure [2].

#### 3.2.5 AFM

AFM images (Fig. 2(B)) demonstrated that all TFP samples possessed a typical chain structure. The branched intertwining of these samples was evident, as their heights were significantly greater than that of a single polysaccharide chain (0.1–1 nm) [29]. This observation was further supported by the cluster conformations and mountain shape in the corresponding 3D AFM images, where abundant hydroxyl groups on the polysaccharide chains engaged in hydrogen bonds, generating strong inter- and intramolecular interactions that contributed to the aggregation. In accordance with their higher M_w_, EEP (125–167 nm, 16.1–27.7 nm) showed considerably greater chain width and height than UEP (83–125 nm, 2.6–7.1 nm) and UAEP (80–120 nm, 1.1–5.6 nm), accompanied by the presence of regular and irregular aggregate lumps [25], [30]. Ultrasound treatment dispersed the intertwined polysaccharide structure, leading to the degradation of long chains and consequent reductions in both width and height. The combined application of ultrasound and cellulase further facilitated the disruption of the main chain of the polymer and multi-branched structure, ultimately yielding short linear fragments and simple branched structures.

#### 3.2.6 Analysis of Congo red experiment

The Congo red experiment is a widely employed method for detecting triple helix structures in polysaccharides. The underlying principle is that a polysaccharide possessing a triple helix conformation can form a complex with Congo Red dye under alkaline condition, resulting in a red shift in the maximum absorption wavelength (λ_max_) relative to pure Congo red [31]. Fig. 2(C) showed the λ_max_ values of UAEP, UEP, EEP mixed with Congo Red over the NaOH concentration range of 0–0.5 mol/L. All three TFP samples exhibited a red shift following complex formation, especially UAEP and UEP, suggesting the presence of triple helix structures in these TFPs [18]. This result was consistent with earlier findings that *Scorias spongiosa* polysaccharides with triple-helix conformations exhibited a red shift within a certain alkaline concentration range [32]. Notably, UAEP and UEP exhibited a higher red shift compared to the EEP, suggesting that ultrasonication might be beneficial for preserving the triple helix conformation of TFPs.

### 3.3 The functional properties of TFPs

#### 3.3.1 Thermodynamic stability analysis of TFPs

**Fig. 3:**
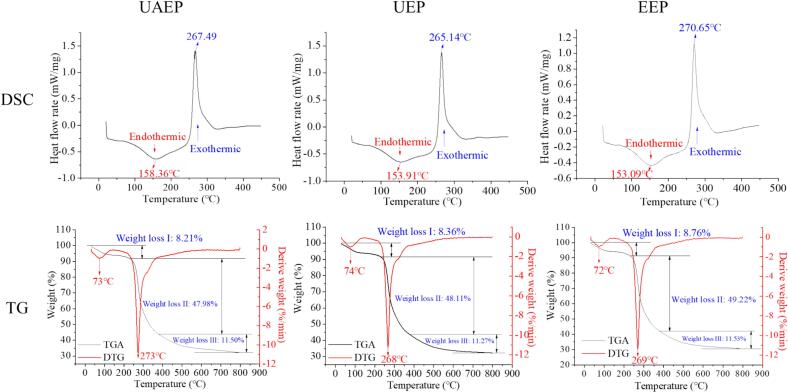
Thermal analyses of different TFPs.

The thermodynamic properties of TFPs were characterized by differential scanning calorimetry (DSC) and thermogravimetric analysis (TGA) (Fig. 3). The DSC curves showed endothermic and exothermic peaks between 20 and 450℃. Endothermic transitions due to bound water evaporation occurred at 86.85–203.71℃ (UAEP), 85.20–202.01℃ (UEP) and 81.47–196.83℃ (EEP). Exothermic peaks corresponding to polysaccharide degradation appeared at the range of 241.21–326.84℃, 234.29–326.29℃, and 243.76–336.41℃, respectively. Accordingly, the glass transition temperatures (T_g_) were 158.36, 153.91 and 153.09℃, while the melting temperatures (T_m_) were 267.69, 265.14, and 270.65℃, respectively. Although T_m_ showed minor variations among the three samples, UAEP displayed a significantly higher ΔH_m_ (Table 2), indicating a broad melting range and superior thermal stability.

**Table 2:** Thermal properties of TFPs obtained from DSC.

Samples	T_g_ (℃)	ΔH_g_ (J/g)	T_m_ (℃)	ΔH_m_ (J/g)
UAEP	158.36 ± 1.35^a^	13.51 ± 0.35^a^	267.49 ± 2.88^b^	74.25 ± 1.18^a^
UEP	153.91 ± 1.61^b^	10.72 ± 0.12^b^	265.14 ± 3.31^b^	64.95 ± 1.24^b^
EEP	153.09 ± 1.32^b^	10.65 ± 0.23^b^	270.65 ± 2.05^a^	58.59 ± 1.53^c^

Note: different letters in the same column represent significant differences (*p <* 0.05).

The thermogravimetric (TG) and differential thermogravimetric (DTG) curves depicted three distinct weight loss stages during heating from 30 to 800°C. The first stage (30–230℃) corresponded to bound moisture loss, with weight losses of 8.76% (UAEP), 8.36% (UEP) and 8.21% (EEP), respectively [4]. The second stage (230–380℃) involved a rapid weight loss due to the thermal decomposition, chain fracture and hydrogen bonds breakage, with losses of 49.22% (UAEP), 48.11% (UEP), and 47.98% (EEP) [33]. The third stage (380–800℃) showed slower weight loss from oxidation and thermal depolymerization of carbonaceous residues, with final residues of 11.53% (UAEP), 11.27% (UEP) and 11.50% (EEP) [34]. Notably, UAEP had a higher maximum decomposition temperature (273℃), implying enhanced thermal stability. In summary, all TFP samples, particularly UAEP, demonstrated good thermal stability and an excellent potential for heat processing application in heat processing in the food industry.

#### 3.3.2 Rheological properties

**Fig. 4:**
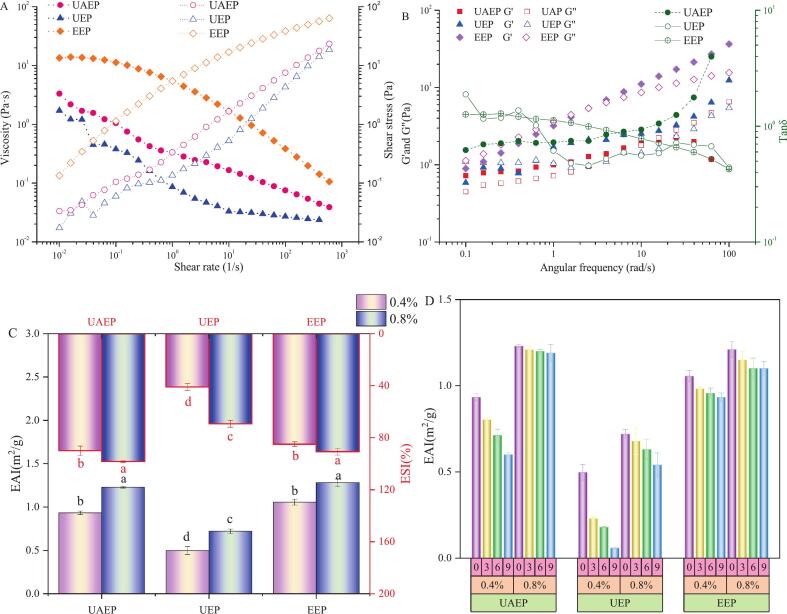
Rheological properties (A and B), emulsifying properties at different concentrations (C) and stored for different days (D) of different TFP samples. Note: different letters in Fig. 4(C) indicate significant differences (*p* < 0.05).

As observed in Fig. 4(A), the viscosity of TFPs gradually declined with an increase in shear rate, characteristic of pseudoplastic shear-thinning behavior. This phenomenon could be attributed to shear-induced disruption of chain entanglements and reorientation of polysaccharide chains along the flow direction. Among the three samples, EEP illustrated the highest viscosity, confirming the higher M_w_ led to higher viscosity [35]. Although UEP had a higher M_w_ than UAEP, its lower viscosity suggested that side chain length and quantity might also significantly influence viscosity [36]. In addition, the viscosity data were subsequently fitted to the Power-law model, and the resulting parameters are summarized in Table 3. According to all the values of R^2^ (>0.9) and n (0 < n < 1), it could be concluded that the results were well fitted by the Power-law model as pseudoplastic fluids [37]. Besides, with increasing shear rate, shear stress gradually increased. Consistent with the viscosity results, EEP exhibited higher shear stress, particularly at higher shear rates, indicating that EEP possessed greater resistance to flow and stronger intermolecular interactions compared to UAEP and UEP.

**Table 3:** Parameters fitted by Power-Law model.

Samples	K/Pa·s^n^	*n*	R^2^
UAEP	0.2936 ± 0.0245^b^	0.4911 ± 0.0211^b^	0.9841
UEP	0.0834 ± 0.0146^c^	0.3428 ± 0.0426^c^	0.9677
EEP	5.1411 ± 0.3642^a^	0.7430 ± 0.0206^a^	0.9282

Note: different letters in the same column represent significant differences (*p <* 0.05).

Fig. 4(B) illustrates the frequency-dependent viscoelastic behavior of TFPs, expressed as storage modulus (G′), loss modulus (G″) and loss factor (tanδ = G“/G'). UEP and EEP displayed liquid behavior with sticky characteristic at relatively low frequencies (G'<G”, tanδ > 1) and a gel behavior with elastic characteristic at relatively high frequencies (G'>G”, tanδ < 1). In contrast, UAEP exhibited the opposite frequency response. This discrepancy was likely due to the differences in the degree of chain disentanglement among the samples. Furthermore, because of their higher M_w_, EEP and UEP showed lower crossover frequencies, reflecting a more strongly entangled polymer network and reduced chain mobility [8].

#### 3.3.3 Emulsifying properties

As shown in Fig. 4(C), UAEP and EEP illustrated a higher EAI than UEP at both 0.4% and 0.8% (w/v) concentrations, with no significant difference between themselves. This enhanced performance might be partially associated with their higher protein content compared to UEP, which could promote more effective absorption at the oil–water interface [38]. In addition, the relatively higher viscosity of UAEP and EEP might be a contributing factor to their enhanced EAI, possibly related to their resistance against gravity and reduced collision probability of droplets [37], [38]. Interestingly, UAEP exhibited higher ESI than EEP, despite its lower M_w_. This suggested that a moderate reduction in M_w_ might be favorable for emulsion stabilization. This is supported by SEM and AFM results, which collectively demonstrated that UAEP possessed a loosely packed, less aggregated structure with high surface area, favorable for enhanced hydration and interfacial adsorption capacity. Furthermore, our findings are in agreement with a previous study, which reported that appropriate ultrasound (270 W, 25 min, in an ice-water bath) boosted the emulsifying ability of soluble soybean polysaccharides by reducing particle size, thereby promoting adsorption at the oil–water interface [39]. After 9 days of storage at 4℃, the EAI of all TFP emulsions (0.4% and 0.8%, w/v) declined, especially at the lower concentration (Fig. 4(D)). During storage, UAEP and EEP maintained significantly higher EAI values than UEP (*p* < 0.05), and increased concentration generally improved emulsifying performance. Notably, at 0.8% concentration, the EAI of UAEP gradually exceeded that of EEP over time, suggesting that emulsions prepared with UAEP exhibited better EAI retention during storage, which might be an indicator of superior long-term stability.

### 3.4 PCA

**Fig. 5:**
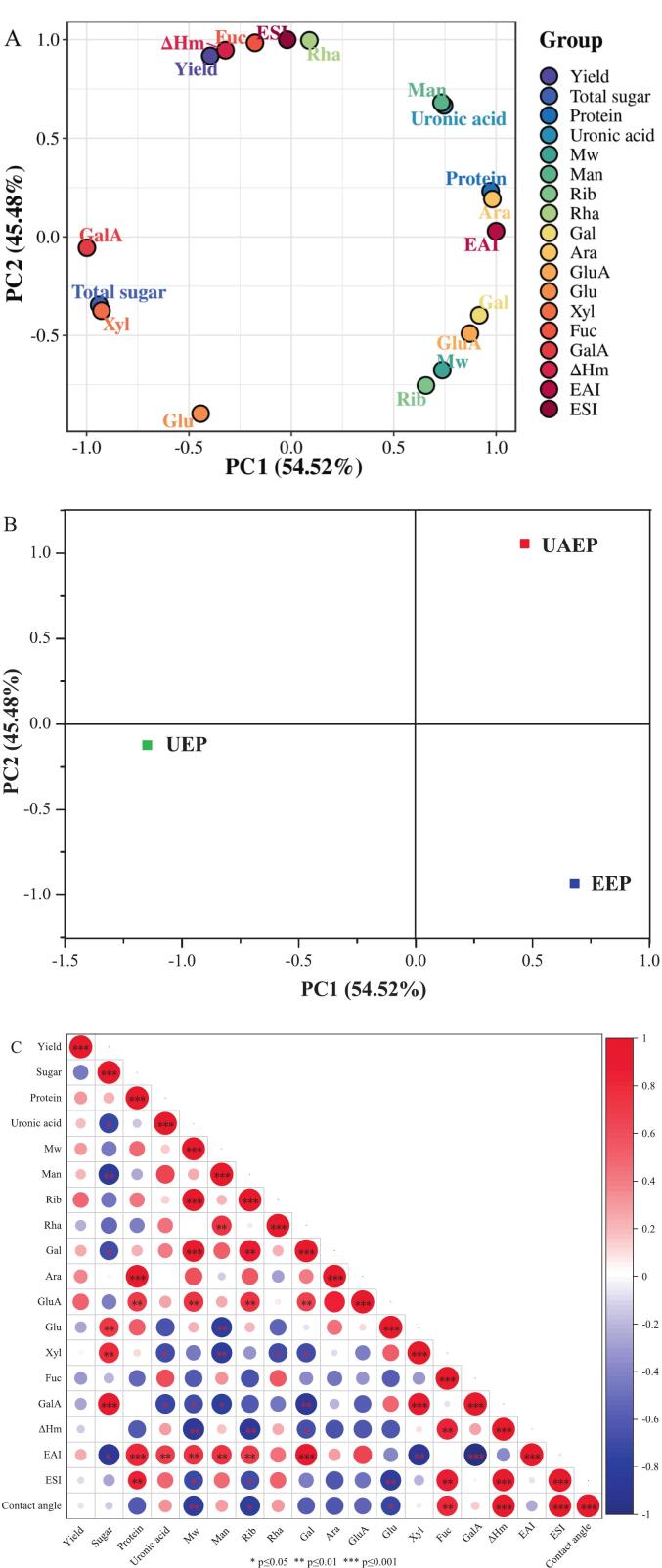
Principal component dispersion map of TFPs with different structural and functional properties (A), PCA plot of TFPs extracted by different methods (B), Pearson correlation matrix of structural and functional properties of TFPs (C).

As a conventional multivariate statistical method, PCA can simplify complex multidimensional data into visualizable components, making it much easier to interpret differences among samples. This method proved particularly valuable for distinguishing TFP samples by clustering them according to extraction methods. The total variation between the extraction methods was fully explained by Principal Component 1 (PC1, 54.52%) and Principal Component 2 (PC2, 45.48%). From Fig. 5(A), the negative correlation of PC1 with yield and total sugar content but positive correlation with M_w_ and certain monosaccharides (e.g., Man, Rha) indicated that UEP and EEP might preserve higher molecular weight chains but at the expense of extraction efficiency and possibly functional diversification. Furthermore, the association of ΔH_m_ and Fuc with PC2 for UAEP hinted at enhanced thermal stability and specific structural motifs that might contribute to its improved functional performance [40]. The distinct positioning of UAEP suggested that the synergistic effect of ultrasound and enzymatic treatment not only increased yield but also modified the polysaccharide’s chemical and physical properties in a way that optimized its emulsifying potential [41]. This aligned with the earlier findings that UAEP was characterized by a more porous microstructure and shorter chains, which likely facilitated better interfacial adsorption.

According to Fig. 5(B), the clear separation of UAEP from UEP and EEP along PC2 underscored the unique structural and functional signature imparted by the combined extraction process. The positive loading (such as uronic acid and protein) and negative loading (such as decreased M_w_) of key emulsification-related parameters on PC2 for UAEP suggested that ultrasound-assisted enzymatic extraction enhanced the presence of these functional components, which were known to improve interfacial activity and emulsion stability [42]. Specifically, moderate M_w_ could adsorb more rapidly at the oil/water interface [38], while uronic acid groups contributed to electrostatic stabilization [40]. In the PCA study by Olawuyi et al. [41] on okra polysaccharides, ultrasound-assisted enzymatic extraction was associated with high acetylation, high emulsion stability and high antioxidant activity, as well as low molecular weight and low viscosity, which clearly distinguished these polysaccharides from those obtained by hot-water, enzyme-assisted and ultrasonic-assisted extraction. Similarly, our PCA results revealed that UAEP exhibited comparable characteristics, including superior emulsion stability, thermal stability, lower molecular weight, and a more porous microstructure.

In summary, PCA not only validated the efficacy of ultrasound-assisted enzymatic extraction in producing functionally distinct TFP but also provided actionable insights into the key structural drivers (e.g., M_w_, uronic acid) of emulsification performance, further supporting the advantage of ultrasound-assisted enzymatic extraction over ultrasound-alone or enzyme-alone methods.

### 3.5 Correlation analysis

The structure–property relationship in polysaccharides is governed by their structural characteristics, specifically, the M_w_, monosaccharides composition, and types of chemical groups are critical determinants of their functionality [43]. Therefore, a Pearson correlation analysis was conducted to examine the relationships between structural characteristics and functional properties of TFPs extracted by different methods, with color intensity proportional to the magnitude of the correlation coefficient [44]. Positive correlations are depicted by red regions, while negative correlations are represented by blue areas; deeper color saturation corresponds to stronger correlation strength between indicators.

As displayed in Fig. 5(C), the EAI and ESI were related to Man, Rib, Gal, Glu, Fuc and total sugar, implying the presence of neutral sugar side-chains might exert a profound influence on the emulsifying properties of the TFPs, which was consistent with the findings of Cao et al. [43]. Furthermore, EAI demonstrated a positive correlation with M_w_ (*p* < 0.01), whereas ESI exhibited an inverse relationship with M_w_ (*p* < 0.05). Although a higher molecular weight generally strengthened the steric hindrance of polysaccharides and thus helped prevent droplet aggregation, the ultrasound-induced reduction in M_w_ and particle size, together with the conformational rearrangement of polysaccharide chains, ultimately contributed to improved emulsion stability [45]. Furthermore, significant correlations were observed between protein content and EAI (*p* < 0.001) and ESI (*p* < 0.01), confirming the importance of protein in emulsifying properties [46]. Additionally, contact angle exhibited a highly significant positive correlation with ΔH_m_ and ESI (*p* < 0.001), while demonstrating a significant negative correlation with M_w_ (*p* < 0.01). These correlations suggested that surface wettability, as reflected by contact angle, was associated with the thermal stability and emulsifying performance of TFPs [47].

These results further reinforced the conclusion that ultrasound-assisted enzymatic extraction effectively influence the structural properties of TFP to optimize its emulsifying functionality by reducing M_w_, modifying monosaccharide composition, and enhancing hydrophobicity, highlighting the critical role of extraction methods in modulating polysaccharide structure and performance.

### 3.6 Application in almond-based milk

#### 3.6.1 Stability coefficient (R)

Plant-based protein milk is a colloidal liquid system typically formulated from plant-derived materials such as nuts, seeds, or legumes (such as walnuts, almonds, soybeans). Incorporating TFP into plant‑based protein milk not only improved emulsion stability through its interfacial activity but also enhanced the nutritional value of the milk system.

**Fig. 6:**
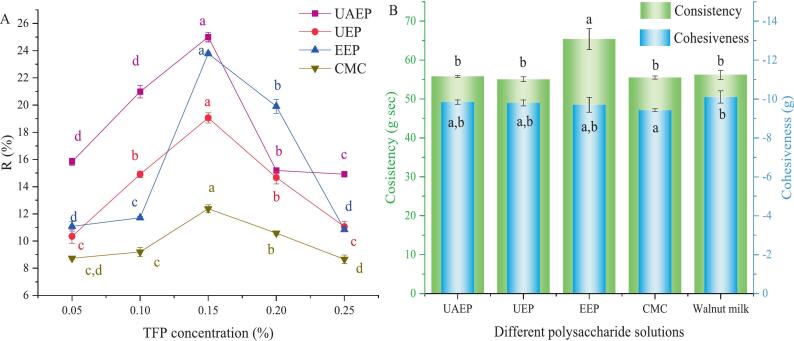
Effect of different concentrations of polysaccharide solutions on the stability coefficient (A), consistency and cohesiveness (B) of almond-based milk. Note: different letters indicate significant differences (*p <* 0.05).

The effect of different concentrations of polysaccharides on the R value of almond-based milk was shown in Fig. 6(A). At different concentrations, UAEP demonstrated the overall best stabilizing effect, exhibiting the highest R value at each concentration point except at 0.2% (w/v). Although UAEP and EEP showed comparable stability at 0.15%, UAEP performed slightly better at both lower and higher concentrations. This superior performance of UAEP could be attributed to the lower M_w_ and shorter linear chain segments, which promoted the formation of a uniform, moderately viscoelastic three-dimensional network in the continuous phase through chain entanglement and hydrogen bonding [48]. This network stabilized the emulsion sterically and by inhibiting droplet migration. In contrast, the higher M_w_ of EEP might promote localized gelation rather than a homogeneous network, while the denser structure of UEP likely limited its network-forming ability.

Notably, all three TFP samples provided significantly better stability than CMC across all tested concentrations. This was likely because TFPs, as naturally extracted complexes, contained protein fragments and hydrophobic regions that imparted amphiphilicity, enabling rapid and strong adsorption at the oil–water interface to form a cohesive elastic film [49]. In contrast, CMC, as a highly hydrophilic anionic cellulose derivative, exhibited weak interfacial activity and primarily stabilized the system through thickening effects.

#### 3.6.2 Texture analysis

The texture profile, specifically consistency and cohesiveness, of almond‑based milk containing different TFP samples and CMC was evaluated. Consistency reflects the resistance to flow, whereas cohesiveness indicates the internal binding strength of the product, with more negative cohesiveness values generally corresponding to slower spreading on the tongue. A commercial walnut protein‑based milk was included as a textural benchmark. This comparison is justified because walnut milk is a well‑established commercial product in the plant‑based milk category with desirable mouthfeel and consumer acceptance. Moreover, according to the USDA FoodData Central database [50], both almond and walnut are tree nuts with comparable macronutrient compositions: both are rich in unsaturated fatty acids (approximately 50–65% fat) and contain moderate levels of protein (approximately 15–21%). This similarity made textural comparison meaningful despite differences in raw materials. The goal was to assess whether almond milk with added TFP could achieve textural attributes comparable to an existing commercial product, thereby demonstrating its practical potential as a natural hydrocolloid. In terms of consistency, almond-based milk with UAEP (55.81 g·sec) and UEP (55.05 g·sec) exhibited values closest to those of the CMC-containing sample (55.51 g·sec) and the commercial walnut protein-based milk (56.23 g·sec), demonstrating their ability to mimic the mouthfeel of commercial products (Fig. 6(B)). In contrast, milk containing EEP showed a significantly higher consistency (65.41 g·sec), likely due to its higher M_w_ or distinct rheological behavior, imparting an undesirably thick or viscous sensation. Regarding cohesiveness, all tested samples fell within a similar range (approximately ‑9.8 to ‑10.1 g), suggesting that polysaccharide addition did not substantially alter the structural integrity or oral breakdown behavior. Notably, UAEP (‑9.84 g) displayed a cohesiveness value closest to that of the commercial milk (‑10.10 g). Collectively, these results demonstrated that UAEP could effectively serve as a natural hydrocolloid in plant‑based milk, providing both suitable consistency and desirable mouthfeel characteristics. Its ability to match key commercial benchmarks in texture confirmed its potential as a dual‑function ingredient that stabilized emulsions while modulating product rheology.

## 4 Conclusions

Ultrasound-assisted enzymatic extraction significantly enhanced the yield of *Tremella fuciformis* polysaccharide (UAEP) to 28.19%, compared to 18.79% and 11.23% for ultrasound-alone (UEP) and enzyme-alone (EEP) extraction, respectively. UAEP exhibited a reduced M_w_, increased uronic acid content, and a more porous microstructure, while preserving the primary monosaccharide composition and chain conformation. Multivariate analyses distinguished UAEP from other TFP samples and confirmed that the structural distinctions induced by combined ultrasound and enzymatic treatment were closely linked to its enhanced emulsifying properties. Importantly, UAEP was successfully incorporated into almond-based milk, where it effectively improved physical stability and achieved textural properties comparable to a commercial plant-based protein milk. Collectively, these findings demonstrate that ultrasound-assisted enzymatic extraction is an efficient strategy for producing TFPs with specific structural and functional properties, positioning them as promising hydrocolloids for plant-based food applications. However, further studies are warranted to evaluate the scalability of this approach and to assess the long-term stability and sensory attributes of UAEP-fortified products under real-world storage conditions.

## CRediT authorship contribution statement

**Furong Hou:** Writing – review & editing, Writing – original draft, Investigation, Funding acquisition, Formal analysis, Conceptualization. **Shasha Song:** Visualization, Methodology, Investigation, Funding acquisition. **Jie Mi:** Methodology, Data curation. **Yansheng Wang:** Resources, Project administration. **Hao Dong:** Visualization. **Wenliang Wang:** Resources, Project administration.

## Declaration of competing interest

The authors declare that they have no known competing financial interests or personal relationships that could have appeared to influence the work reported in this paper.
